# Development of a rapid and economic ***in vivo*electrocardiogram platform for cardiovascular drug assay and electrophysiology research in adult zebrafish**

**DOI:** 10.1038/s41598-018-33577-7

**Published:** 2018-10-30

**Authors:** Min-Hsuan Lin, Huang-Cheng Chou, Yu-Fu Chen, Wangta Liu, Chi-Chun Lee, Lawrence Yu-Min Liu, Yung-Jen Chuang

**Affiliations:** 1 Department of Medical Science & Institute of Bioinformatics and Structural Biology, National Tsing Hua University, Hsinchu, 30013 Taiwan; 2 Department of Electrical Engineering, National Tsing Hua University, Hsinchu, 30013 Taiwan; 3 Department of Medical Science, National Tsing Hua University, Hsinchu, 30013 Taiwan; 4 Department of Biotechnology, College of Life Science, Kaohsiung Medical University, Kaohsiung, 80708 Taiwan; 5 Division of Cardiology, Department of Internal Medicine, Hsinchu Mackay Memorial Hospital, Hsinchu, 30071 Taiwan

## Abstract

Zebrafish is a popular and favorable model organism for cardiovascular research, with an increasing number of studies implementing functional assays in the adult stage. For example, the application of electrocardiography (ECG) in adult zebrafish has emerged as an important tool for cardiac pathophysiology, toxicity, and chemical screen studies. However, few laboratories are able to perform such functional analyses due to the high cost and limited availability of a convenient *in vivo* ECG recording system. In this study, an inexpensive ECG recording platform and operation protocol that has been optimized for adult zebrafish ECG research was developed. The core hardware includes integration of a ready-to-use portable ECG kit with a set of custom-made needle electrode probes. A combined anesthetic formula of MS-222 and isoflurane was first tested to determine the optimal assay conditions to minimize the interference to zebrafish cardiac physiology under sedation. For demonstration, we treated wild-type zebrafish with different pharmacological agents known to affect cardiac rhythms in humans. Conserved electrophysiological responses to these drugs were induced in adult zebrafish and recorded in real time. This economic ECG platform has the potential to facilitate teaching and training in cardiac electrophysiology with adult zebrafish and to promote future translational applications in cardiovascular medicine.

## Introduction

Electrophysiology is a unique component of biomedical science capable of investigating the electrical properties of an individual cell, organ, or complete organism in the context of physiology. Regarding the heart, the process of recording cardiac electrical activity is known as electrocardiography (ECG). Generally, multiple electrodes are attached to specific sites on test subject’s body surface to record the electrical signals generated from the cardiac conduction system, which represent the polarization and depolarization of cardiac muscle tissues. These signals can then be interpreted to reveal normal conduction or specific diseases that result in cardiac arrhythmia. Thus, we can recognize a health condition emerging in real time by examining the *in vivo* ECG recording and identifying relevant electrophysiological alterations in the heart.

Since ECG reflects *in vivo* cardiac function, current FDA (Food and Drug Administration) regulation requires pharmaceutical companies to perform animal ECG assessments for cardiac toxicity when developing a new drug at the preclinical stage. These assessments are required to avoid adverse drug effects on the human heart, such as arrhythmia or heart failure. Therefore, there is an important need to develop efficient electrocardiogram methods for predictive assays of cardiotoxicity in animal models.

Given the rapid advancement in gene editing technology, spontaneous heart disease models have become easier to generate in zebrafish, as zebrafish are an accessible model organism for genetic modification and crossbreeding. Ideally, an animal heart disease model should be easily manipulated, be reproducible, exhibit representative characteristics of human pathophysiology and be ethically sound. The low cost and easy manipulation of zebrafish for cardiovascular research make it an increasingly popular animal model to be considered for ECG studies^1^.

Zebrafish has only two chambers in its heart, but the cardiac electrophysiology of zebrafish is highly similar to that of the four-chambered heart of human. Cardiac action potentials (AP) in both human and zebrafish are generated by the movement of ions through the transmembrane ion channels in cardiac cells^2^. It is noteworthy that ion channels dominating the AP upstroke in zebrafish are well conserved in human. Consequently, zebrafish heart also presents a distinct P-wave, QRS-complex, and T-wave on ECG recording, all of which are comparable to the ECG features of human^3,4^. However, zebrafish ECG is not yet an easily accessible technique. Current zebrafish ECG recordings typically require specialized devices and software, including an amplifier, a bandpass filter, and digitized data-processing software, which collectively come at high cost.

In this paper, we describe the setup of an economic zebrafish ECG system that is based on integration of a ready-to-use electrophysiological recoding kit with custom-made needle electrode probe, which should be highly accessible for most research and teaching laboratories. For general testing of the optimized protocol, we used this system to monitor the cardiac physiological responses of adult zebrafish to common anesthetics and selected antiarrhythmic medications in real time. We anticipate that the devices and protocol described in this study can be established in any laboratory, which would greatly benefit educational and research practices with zebrafish.

## Results

### Constructing the adult zebrafish ECG system

The simple and ready-to-use ECG kit (Ez-Instrument Technology Co., Taiwan) was originally developed for teaching purposes at high-school biology laboratories. The original kit comprised an integrated signal receiver and amplifier, and a packaged software for signal visualization and basic data processing tools. We explored the use of the kit on adult zebrafish ECG for more advanced research applications by re-designing the specialized electrode probe for this new purpose. After reviewing the commercially available electrode probes and published protocols on adult zebrafish ECG^3^, we custom-made a three-needle electrode probe that could be directly connected to the ready-to-use ECG kit for real-time recording of ECG signals on anesthetized adult zebrafish (Supplemental Fig. 1A).

The design of the three-point needle electrode probe integrated a pectoral electrode, an abdominal electrode and a grounding electrode (Supplemental Fig. 1B–C). Each electrode harbored a stainless-steel needle coated with insulating paint on most of its surface to reduce noise from the aquatic environment. The needle head was uncoated to allow a 1-to-1.5 mm exposed area for signal detection, whereas the tail end was welded to the connecting wire and clustered into a 3-pole auxiliary connector. The probe had a plastic holder to secure the stainless-steel needle and connective wire and enable the probe to be fastened onto the micromanipulator (Supplemental Fig. 1C). The ECG system was ready for experiments once the customized electrode probe was connected to the ECG kit and the analysis software was running. In summary, the zebrafish ECG system consisted of a three-needle electrode probe, two micromanipulators to hold the pectoral and abdominal needles, an ECG kit, and a laptop computer preloaded with the provided software.

### ECG recording of adult zebrafish

**Figure 1:**
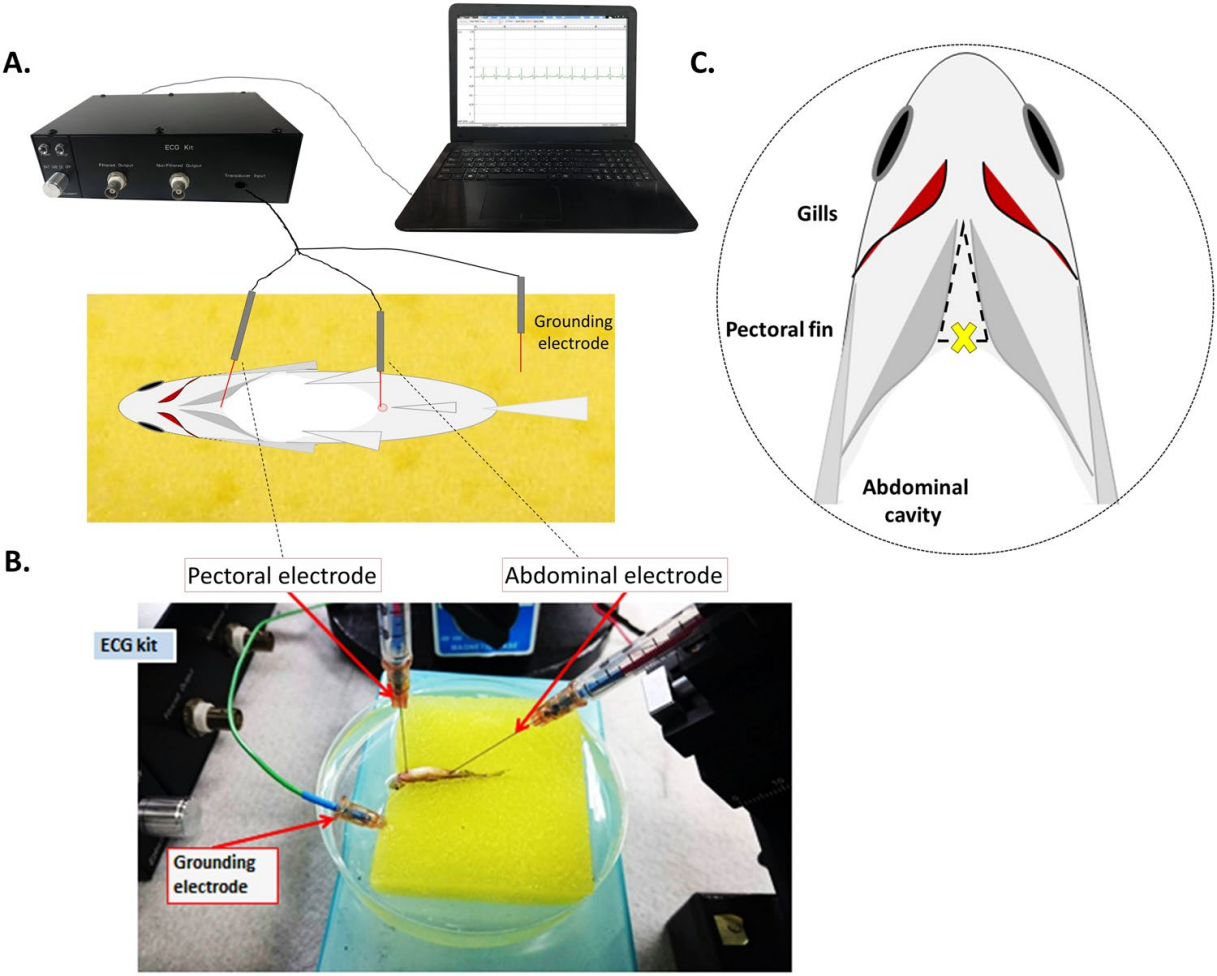
Illustration showing the system setup and the three-needle electrode placement during real-time adult zebrafish ECG recording. The zebrafish is anesthetized and immobilized on the immersed sponge. Two of the electrodes are positioned on micromanipulators. The electrode above the zebrafish’s heart is penetrated through the dermis with a micromanipulator. In this way, the penetration depth of the electrode probe can be measured by reading from the scale on the micromanipulator. ECG kit: The signals from the electrodes are amplified, filtered and converted to digital signals in this black box. (**A**) Scheme of ECG recording system. (**B**) Photograph of the experimental setup and positions of the recording probes. (**C**) Standard location of the pectoral recording probe in *in vivo* system as indicated by a cross in the field of view under a dissecting microscope.

To record adult zebrafish ECG in real time, we referenced previously described protocols to develop an optimized procedure^3^. During ECG recording, the zebrafish was sedated in the anesthetic water bath while wedged into a cleft in a damp sponge to maintain its dorsal side up. A concave triangular section of the sponge was cut away to enable the fish to move its gill opercula during the experiment. The pectoral scales above the heart were removed with sharp tweezers to allow penetration of the electrode needle tip. The field of operation, i.e., the triangular region between the pectoral fins below the head, was monitored under a microscope. With the micromanipulator, the pectoral electrode needle was gently inserted into the thorax to a depth to detect the ECG signal without damaging the heart tissue (Fig. 1C). The abdominal probe was gently inserted into the cloaca (i.e., the posterior anal/reproductive orifice) with a second micromanipulator. The insertion depth for both of the pectoral and abdominal needle electrodes was approximately 1–1.5 mm. The grounding electrode was placed at the corner of the damp sponge as a reference electrode (Fig. 1A,B).

During ECG recoding, it was necessary to adjust the pectoral electrode probe slightly until the P wave, QRS complex and T wave were clearly recognized on the software’s display window. With the tricaine methanesulfonate (MS-222)/isoflurane combination anesthetics described below, the zebrafish could be safely sedated for 30 minutes. The recording time window was adjusted according to the individual assay and drug response. After recording, the zebrafish was immediately transferred to a recovery tank with clean system water.

### Electrocardiography of adult zebrafish

**Figure 2:**
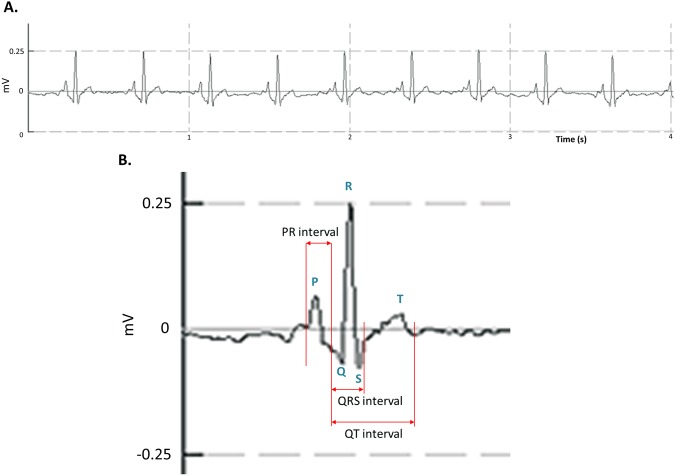
An ECG recording of adult zebrafish with the three-needle electrodes. Regular and distinct P waves, QRS complexes, and T waves can be identified. (**A**) Automatic identification of each heart cycle. The zebrafish raw ECG signal was similar to that in human ECG. The signal includes distinct P waves, QRS complexes, and T waves that can be easily identified. (**B**) Measurement of ECG intervals based on the trace.

The system and procedure described above allowed reliable detection and recording of real-time ECG signals from adult zebrafish. There was no need for additional data fitting or processing. An example of the baseline real-time zebrafish ECG waveform is shown in Fig. 2A, which is highly comparable to the human ECG.

Key features of the zebrafish ECG signal, such as the P wave, QRS complex and T wave, can be easily recognized (Fig. 2B). To establish standard zebrafish ECG parameters, we determined the mean heart rates of wild-type AB zebrafish to be 148 ± 15 beats per minute (bpm). After statistical analysis, we found that in normal, 10- to 12-month-old zebrafish, the average PR interval was 62 ± 4 millisecond (ms), the average QRS interval was 44 ± 3 ms, the average RR interval was 469 ± 54 ms, the average QT interval was 215 ± 43 ms, and the mean HR corrected QT interval (QTc) was 279 ± 60 ms. These results were highly consistent with previous findings^5^, which further demonstrated that this economical ECG system is comparable to more complex ECG systems.

### Electrocardiography of zebrafish under prolonged sedation

Before performing the chemical-induced arrhythmic response assays, we verified the anesthetic effects on zebrafish cardiac physiology using the ECG system. We did so because MS-222, the only FDA-approved anesthetic for fishes, has been shown to affect heart rate in adult zebrafish during sedation. As an alternative anesthetic approach, we used the 140 ppm combined anesthetic formula (70 ppm MS-222 + 70 ppm isoflurane) previously developed in our laboratory, which shows minimal effects on the zebrafish heart rate^6^.

**Figure 3:**
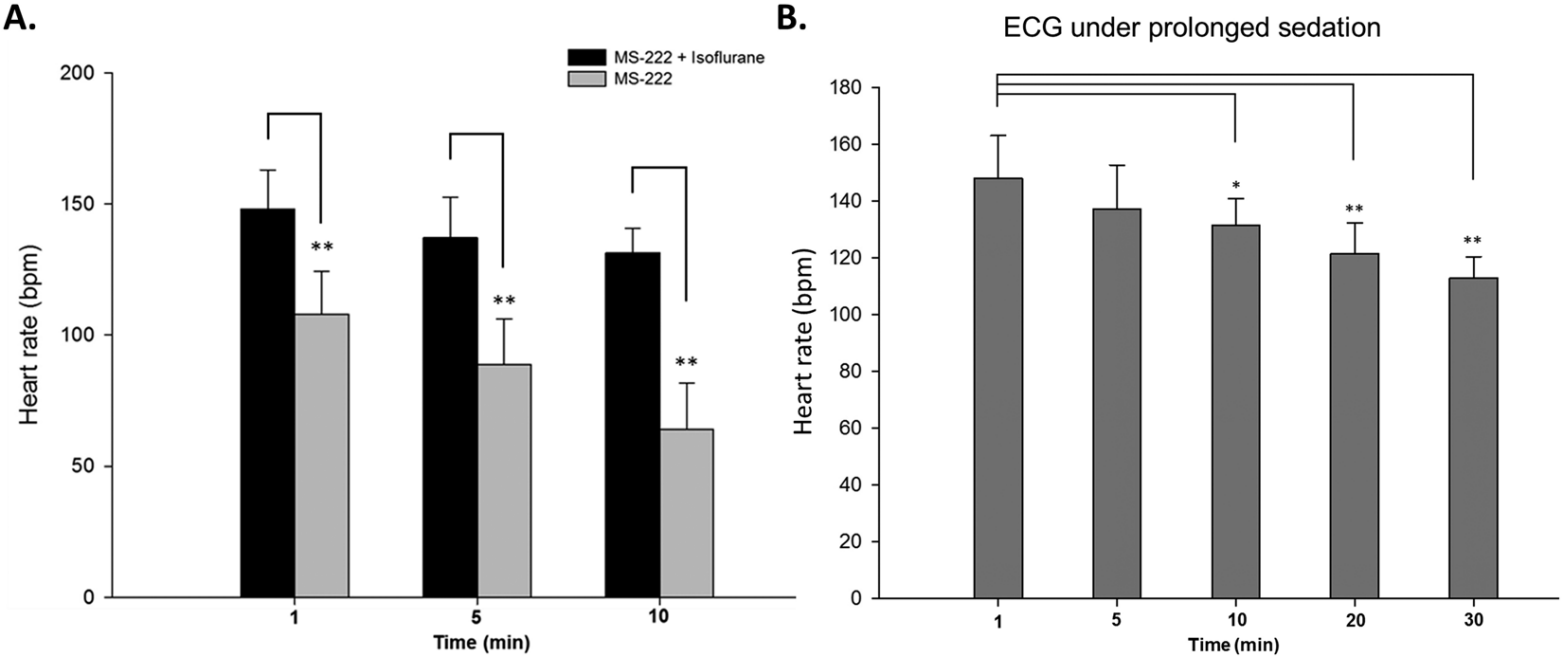
Effects of anesthetics: MS-222 alone and MS-222/isoflurane combination and prolonged sedation with combined formula on heart rate. (**A**) MS-222 reduced the heart rate to 108 ± 16 bpm, whereas the combined formula maintained a normal heart rate of 148 ± 15 bpm. After 10 minutes, the combined-formula group had an average heart rate of 131 ± 19 bpm. Data are presented as the mean ± SD. n = 9 in the MS-222-only group; n = 10 in the combined formula group. (**B**) The combined-formula group maintained a normal heart rate of 148 ± 14 bpm. After 10 minutes, the combined-formula group had an average heart rate 131 ± 9 bpm. Paired *t*-tests showed no significant difference between one minute and 5 minutes and significant differences between the 1- to 10-minute, 20-minute and 30-minute measurements of the combined-formula anesthetic. Data are presented as the mean ± SD. n = 10; *indicates *p* < 0.05, **indicates *p* < 0.001 as determined by paired *t*-tests.

In the MS-222-alone group, the initial heart rate at the first minute was 108 ± 16 bpm, which was significantly lower than that of the combined-anesthetic-formula group, i.e., 148 ± 15 bpm (Fig. 3A). As the sedation time increased, the heart rate in the MS-222 group significantly decreased to 89 ± 17 bpm at 5 minutes, whereas that of the combined-formula group was sustained at 137 ± 16 bpm, which was not statistically different from the rate at one minute.

As expected, after 10 minutes of sedation in MS-222, the heart rate further decreased to 64 ± 18 bpm, whereas the heart rate of the MS-222/isoflurane-combination group remained at 131 ± 19 bpm. These data are consistent with previously published findings^7^. Notably, most of the adult zebrafish in the MS-222-alone group did not recover after 10 minutes of sedation (data not shown). We next focused on analyzing the heart rate variation under prolonged sedation with the combined anesthetic formula (Fig. 3B). After 1, 5, and 10-minute sedation under MS-222/isoflurane anesthesia, the average heart rate was 148 ± 15 bpm, 137 ± 16 bpm and 131 ± 9 bpm, respectively. These data are consistent with previous findings^6^. Prolonged sedation at 20 and 30 minutes yielded an average heart rate of 121 ± 11 bpm and 113 ± 7 bpm, respectively.

Taken together, these data demonstrated that MS-222 may not be a suitable anesthetic for ECG experiments with adult zebrafish. Furthermore, prolonged sedation under the MS-222/isoflurane-combination formula led to a gradual decrease in heart rate beyond 10-minute sedation. We hence recommend that ECG recording experiments be performed using the combined anesthetic formula within the first five minutes; otherwise, the anesthetic effects may result in considerable interference to subsequent analysis.

### Effect of isoproterenol treatment on drug-induced bradycardia

After establishing the optimized ECG assay conditions, we analyzed the effects of common cardiovascular drugs that are frequently used in clinical practice. We started with isoproterenol, which is a nonselective β-adrenergic agonist that is an isopropyl amine analog of adrenaline. Having a well-studied mechanism of action and pharmacological effects on cardiac muscle contractility, isoproterenol can increase the human heart rate and has been prescribed for the treatment of bradycardia^8^.

**Figure 4:**
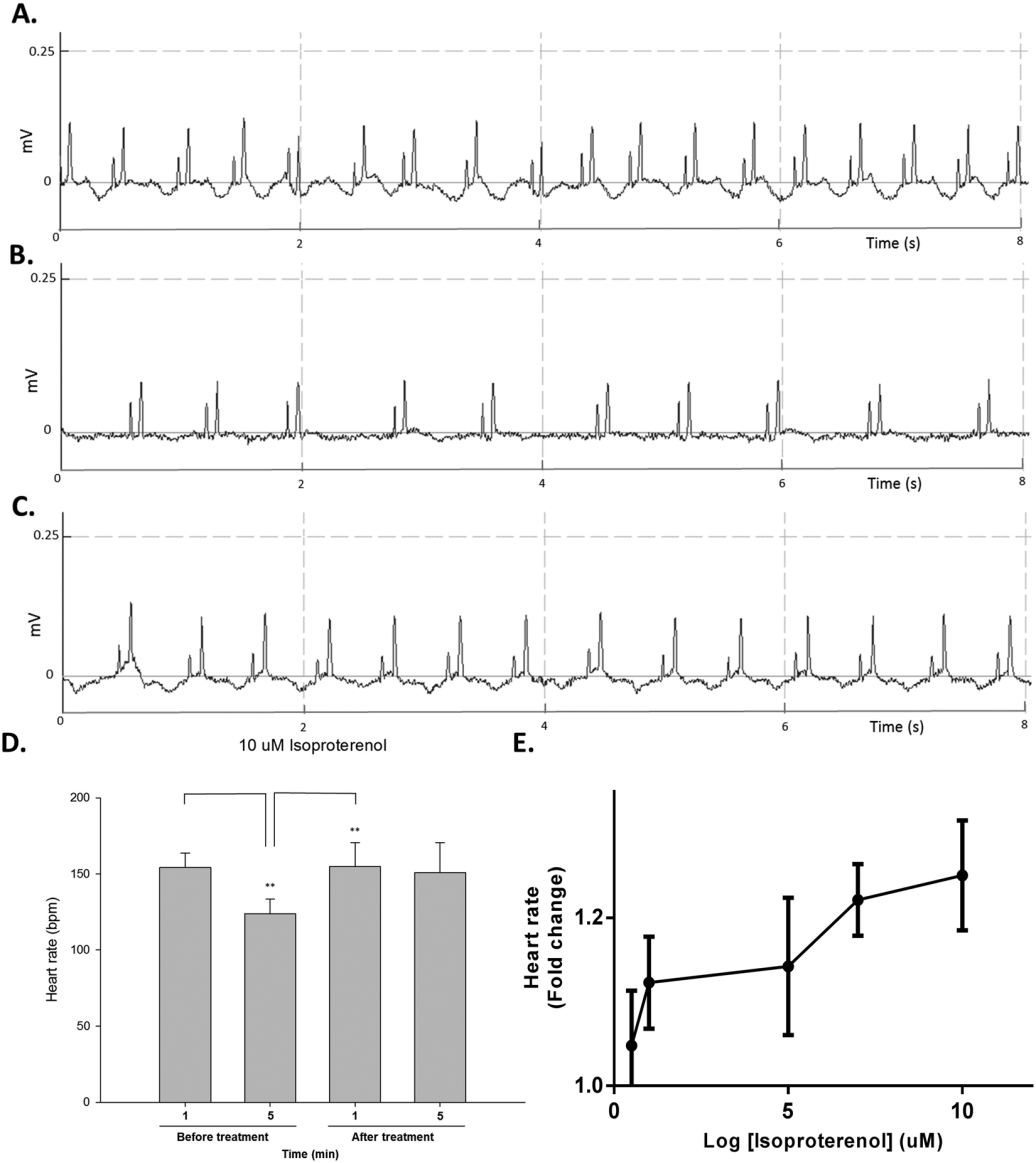
Variation of heart rate during real-time recording of adult zebrafish and the response to isoproterenol. Real-time ECG wave-form changes before and after isoproterenol treatment. (**A**) Raw signal showing normal ECG before isoproterenol injection. (**B**) Extended anesthesia by MS-222 alone to induce bradycardia. (**C**) After isoproterenol treatment, heart rate increased. (**D**) The heart rates were significant reduced after 5-min sedation. Heart rate increased by 1.25 times after 5 μl of 10 μM isoproterenol treatment (n = 6). **Indicates *p* < 0.001 as determined by paired *t*-test. (**E**) Dose-response curve for the effect of isoproterenol on heart rate of zebrafish heart. The fold change of heart rate in response to log of increasing concentration of isoproterenol (0.5, 1, 5, 7.5 and 10 μM, respectively).

Since MS-222 alone was shown in our experiments to reduce heart rate in adult zebrafish, we used this chemical to mimic drug-induced bradycardia and test zebrafish’s response to isoproterenol treatment. Before isoproterenol treatment, the baseline ECG showed the average heart rate to be 159 ± 13 bpm at the first minute. After 5 minutes sedation in 160 ppm MS-222 alone, the heart rate had decreased to 130 ± 16 bpm, as expected. After retro-orbital injection of isoproterenol, a change in heart rate was observed within 60 seconds (Fig. 4A–C). The average heart rate was significantly increased to 155 ± 16 bpm with 5 μl of 10 μM isoproterenol and was higher than that under the MS-222-induced bradycardia condition. We also tested the effect of a lower dose of isoproterenol. We found that isoproterenol had a dose-dependent effect on adult zebrafish heart rate (Fig. 4E). In the group administered with 5 μl of 10 μM isoproterenol, the heart rate was increased by 1.25-fold after isoproterenol injection (Fig. 4D), whereas in the group administered with 0.5 μM isoproterenol, the heart rate only increased 1.04-fold, and 1.12-fold increase in 1 μM group, 1.14-fold increase in 5 μM group and 1.22-fold increase in 7.5 μM group, respectively (Fig. 4E). These results are similar to previous findings reported in human^9^.

### Induction of bradycardia under verapamil treatment

Verapamil is a non-dihydropyridine calcium channel antagonist and a common antihypertensive with an anti-angina effect. However, verapamil can have negative cardiovascular effects in humans, such as abnormal ECG and reduced heart rate^10^. We thus investigated whether these cardiac effects of verapamil could be observed in zebrafish and monitored by ECG in real time.

**Figure 5:**
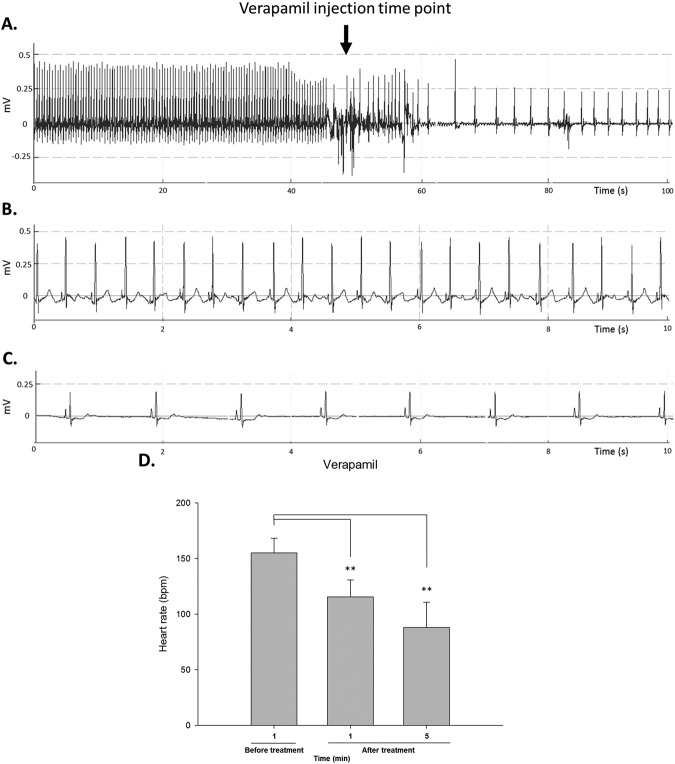
Real-time ECG recording of adult zebrafish and the response to verapamil treatment. (**A**) Simultaneous recording of ECG during retro-orbital injection of verapamil. (**B**) Raw signal showing normal ECG before verapamil injection. (**C**) Bradycardia was observed after verapamil was injected. (**D**) The heart rates were significantly reduced after verapamil injection (n = 7). **Indicates *p* < 0.001 as determined by paired *t*-test.

Before verapamil treatment, the baseline ECG showed the average heart rate to be 155 ± 13 bpm (Fig. 5B). After retro-orbital injection of verapamil, the heart rate decreased to 116 ± 15 bpm within 60 seconds, representing a 25% reduction (Fig. 5C). After 5 minutes, the heart rate had further decreased to 88 ± 22 bpm, a 43% reduction (Fig. 5D). As expected, the results were similar to previous findings^11^, and the effects mimicked the heart rate-lowering effects of verapamil reported in humans^12^. Our results demonstrate that zebrafish and humans have highly conserved action potential responses to verapamil, which confirm the feasibility of using zebrafish ECG for the screening of calcium-channel blocking agents.

### Effects of amiodarone on heart rate, QRS interval, QT interval, PR interval and QTc interval

Amiodarone is a class III anti-arrhythmic drug and has been used to treat and prevent various types of arrhythmia, including ventricular tachycardia and atrial fibrillation^13^. Amiodarone can cause bradycardia and prolong the QT interval^14^. Therefore, we explored whether these cardiovascular effects can be induced in zebrafish.

**Figure 6:**
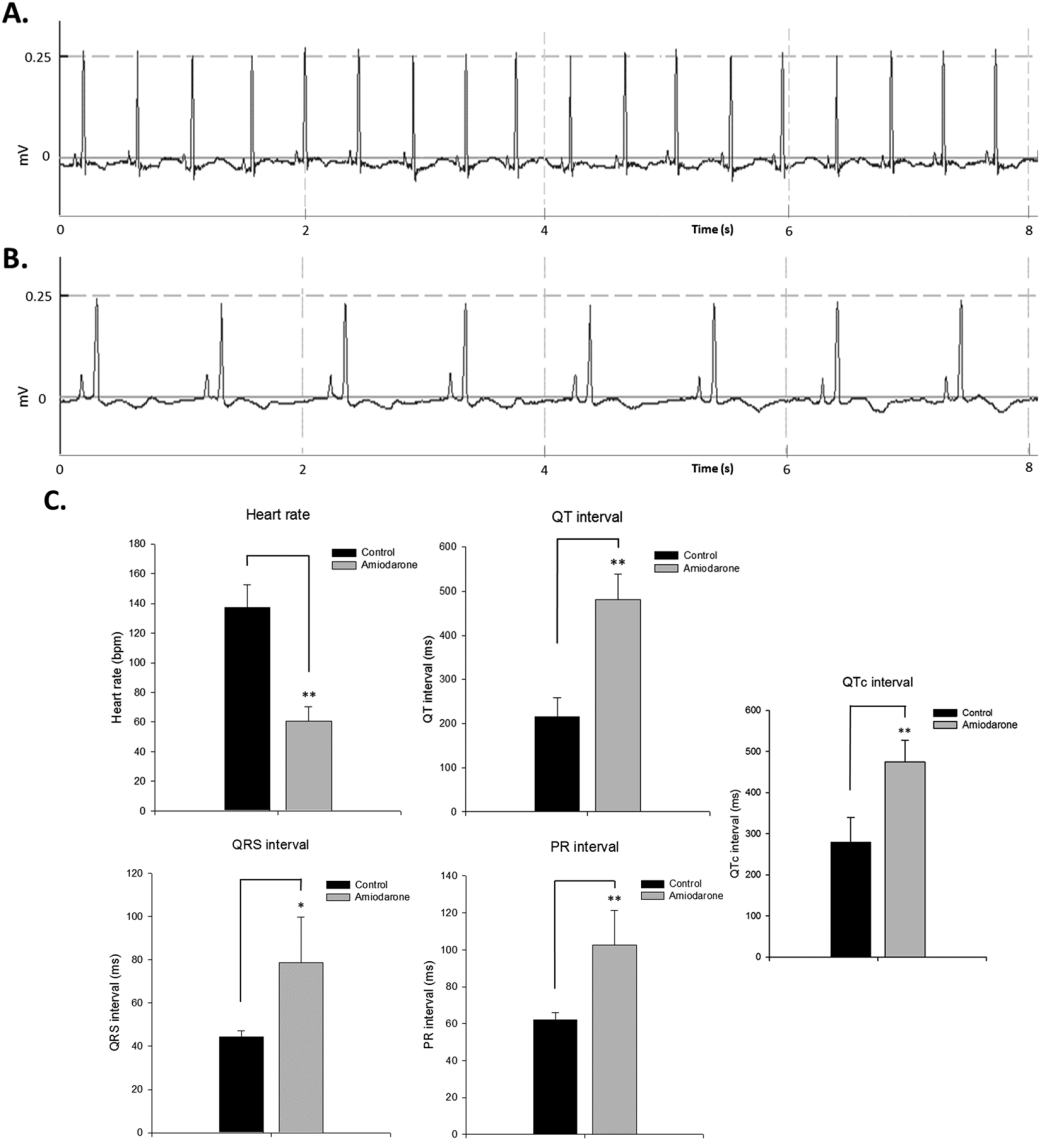
Adult zebrafish ECG features and drug response to amiodarone. ECG waveforms before and after amiodarone treatment are shown. (**A**) Real-time ECG signal before treatment. (**B**) Heart rate was reduced after amiodarone treatment. (**C**) Heart rate, QRS interval, QT interval, PR interval and QTc interval before and after amiodarone treatment. Data are presented as the mean ± SD, n = 12. *Indicates *p* < 0.05, **Indicates *p* < 0.001 as determined by paired *t*-test.

We first immersed the zebrafish in a tank with 100 μM amiodarone in a one-liter water system to mimic acute treatment in human. After one hour of immersion of adult zebrafish in the amiodarone bath, the zebrafish heart rate decreased to an average of 60 ± 10 bpm, which was significantly lower than that of the control group. Analysis of the ECG signals revealed effects of the amiodarone treatment on several ECG features: The QRS interval (79 ± 21 ms) and PR interval (103 ± 19 ms) were found to increase relative to the pretreatment condition values. It is noteworthy that significant QT prolongation was also observed (481 ± 58 ms), and the mean HR corrected QT interval (QTc) was 475 ± 52 ms, which represented a striking 2-fold increase over that of the pretreatment condition (Fig. 6). Bradycardia and QT prolongation indicated the drug’s effects on the ion channels, leading to a decrease in cardiomyocyte excitability and ventricular tachyarrhythmia, respectively. Therefore, these results suggest that zebrafish and humans may have highly conserved ion channels and similar reactions to amiodarone.

### Prolongation of QTc after quinidine treatment

Quinidine is a voltage-gated sodium channel blocker that acts as a class I antiarrhythmic agent (Ia) in the heart to prevent ventricular arrhythmias^15^. Quinidine leads to increase the cardiac action potential duration which also prolongs QT interval and increases the risks of torsade de pointes in human^16^. We then tested these cardiac effects of quinidine in adult zebrafish.

**Figure 7:**
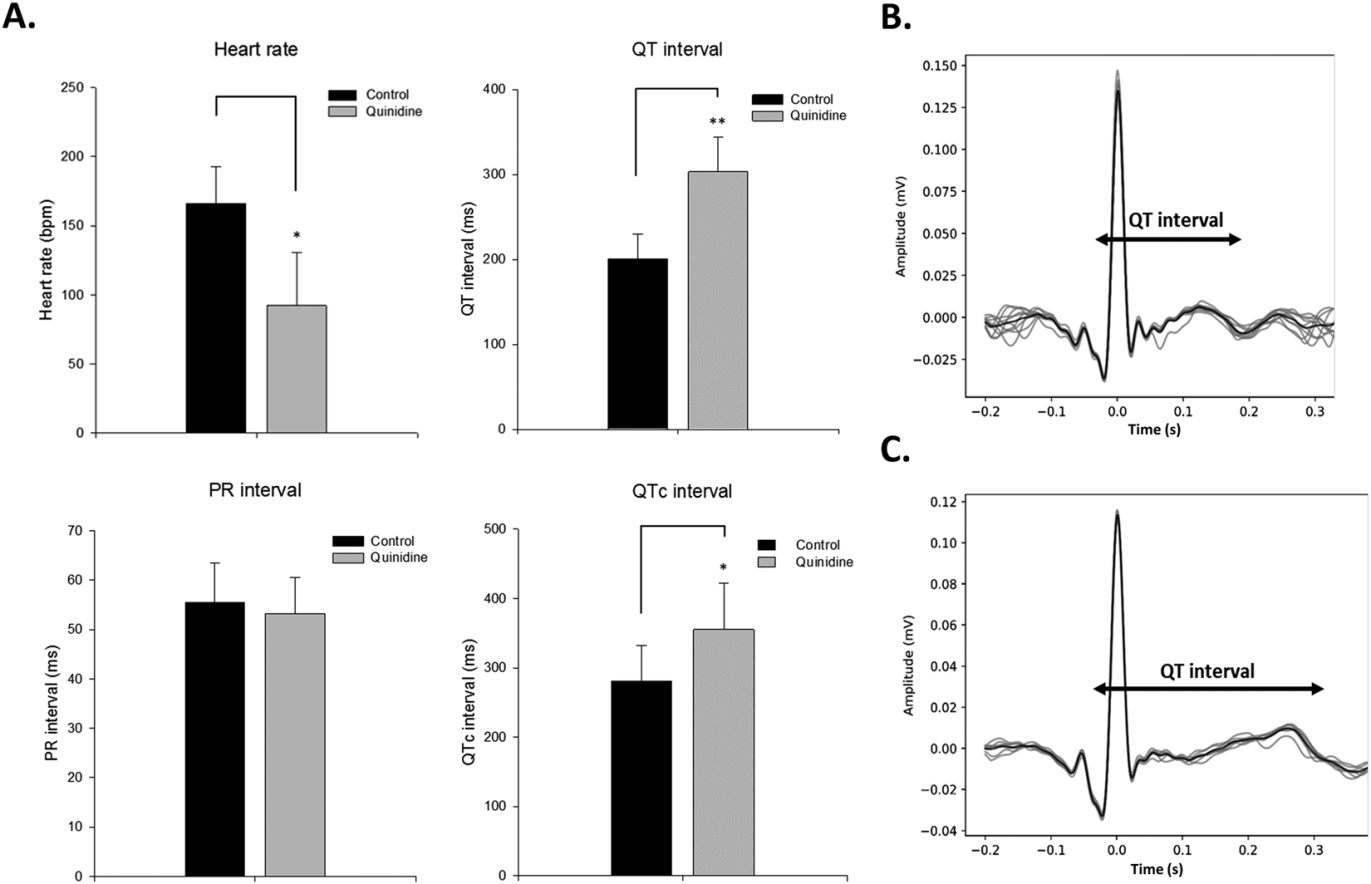
Quinidine reduced heart rate and prolonged QTc interval in adult zebrafish heart. (**A**) Heart rate, QT interval, PR interval and QTc interval before and after 250 μM quinidine treatment. (**B**) Control QT interval before injection of quinidine. (**C**) QT interval was prolonged after quinidine was injected. Data are presented as the mean ± SD, n = 8. *Indicates *p* < 0.05, **Indicates *p* < 0.001 as determined by paired *t*-test.

Before drug treatment, the baseline ECG was at the average heart rate to be 166 ± 27 bpm. After retro-orbital injection of quinidine (250 μM), the heart rate was significantly decreased to 92 ± 39 bpm (Fig. 7A). Specifically, the baseline QT interval was 200 ± 29 ms (Fig. 7B). The QT interval was significantly prolonged to 303 ± 40 ms after post-injection of quinidine (Fig. 7C). The QTc interval prolongation were also seen after drug treatment (from 280 ± 51 to 355 ± 67 ms). PR interval and QRS interval did not have significant change after drug treatment. Thus, zebrafish and humans have highly conserved ion channels and similar ventricular tachyarrhythmia response in the heart.

### Veratridine induce AV-block in adult zebrafish

Veratridine is a plant alkaloid which acts as a neurotoxin and known to depolarize excitable cells by preventing inactivation of voltage-dependent Na^+^ channels^17^. This positive inotropic effect causes an increase in both Na^+^ and Ca^2+^ influx and then increases nerve excitability and cardiac contractility^18,19^. Hence, we used veratridine to simulate gain-of-function on sodium channels to see what proarrhythmic effect could be induced in the adult zebrafish heart.

**Figure 8:**
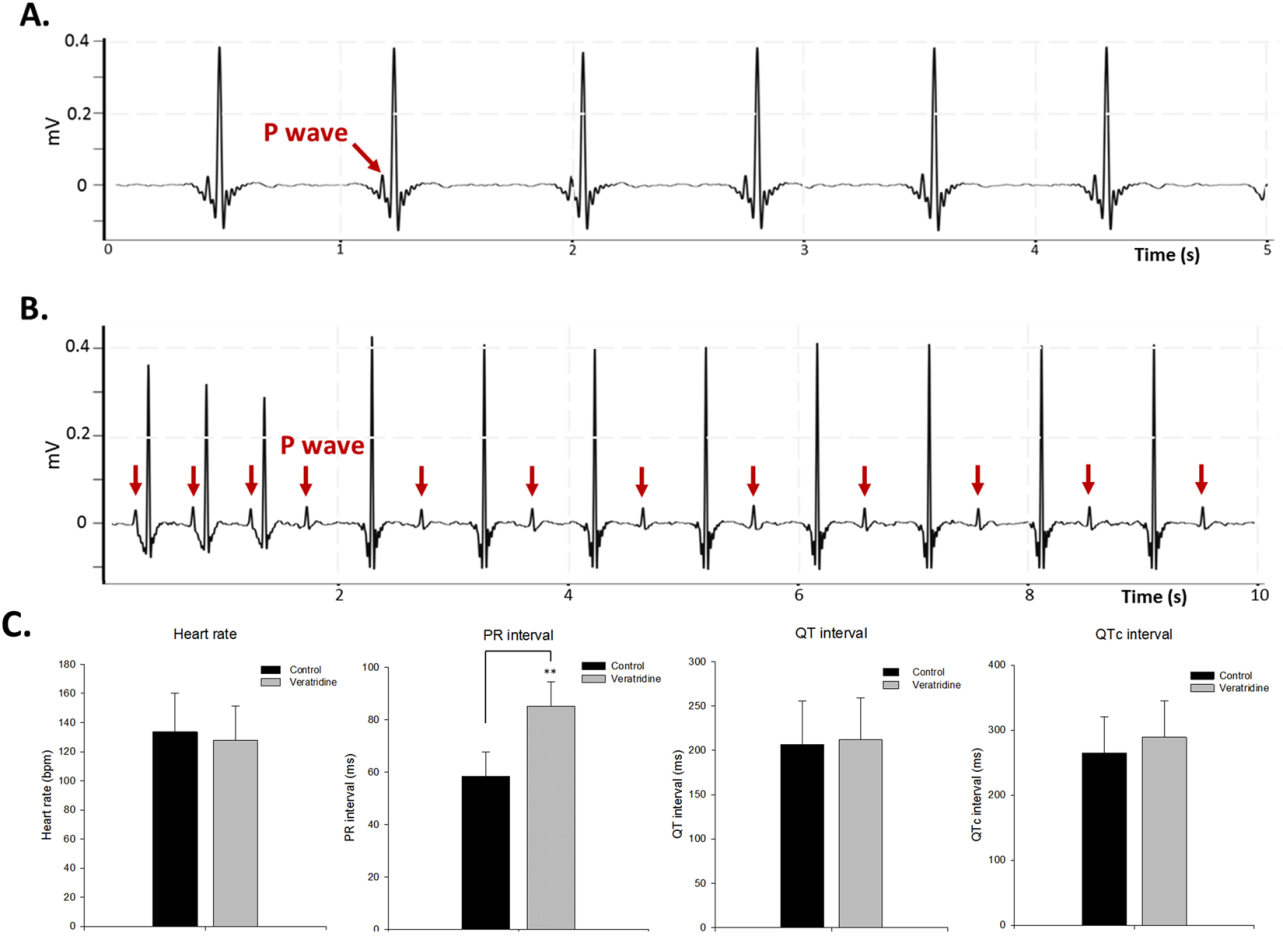
Arrhythmia was induced after veratridine treatment in adult zebrafish. (**A**) Raw signal showing normal ECG before veratridine injection. (**B**) Significant first-degree AV block (prolonged PR interval) was observed within 5 minutes after veratridine (100 μM) was injected. (**C**) Heart rate, PR interval, QT interval and QTc interval before and after veratridine treatment. Data are presented as the mean ± SD, n = 10. **Indicates *p* < 0.001 as determined by paired *t*-test.

Prolonged PR interval (from 58 ± 9 ms to 85 ± 9 ms) was observed in zebrafish injected with veratridine. The Fig. 8B showed first-degree AV block with significant PR interval prolongation at 3 minutes post-injection (8 out of 10 zebrafish). However, heart rate, QT interval, QRS interval and QTc interval did not have any significant change after veratridine treatment (Fig. 8C). These results suggested that the increase in both Na^+^ and Ca^2+^ influx may prolong the PR interval and induce the AV block, but not the QT interval in adult zebrafish heart. These data indicated that veratridine affects the atrioventricular conduction more significantly, however, veratridine may not affect the depolarization and repolarization of the ventricles in the adult zebrafish heart.

## Discussion

In this technical report, we presented a cost-effective, real-time ECG recording system that can be used to monitor ECG signals in adult zebrafish. The easy-to-operate adult zebrafish ECG system could also be extended to pharmacological studies, especially on drugs with proarrhythmic action. We demonstrated that several clinically relevant responses in humans, such as QT prolongation and heart rate variability, can be observed in adult zebrafish. We anticipate that the optimized, straight-forward procedure and highly accessible ECG system shall enhance productivity and learning in both research and teaching laboratories.

To assist other researchers to reproduce our results, we have described in detail on how to set up the ECG system that do not need to be shielded in a Faraday cage during measuring. We also emphasized on optimizing the placement sites of the needle electrode probe (Fig. 1). There are some additional points to consider before initiating ECG recording: (1) When the pectoral electrode probe is inserted too deep into the zebrafish dermis, it can cause a reversed ECG waveform and excessive bleeding of the fish. (2) The p waveform can appear to be sharp and magnified when the pectoral needle probe is moved even slightly toward the right of the center body line. (3) When the pectoral probe was moved toward the left of the center body line, the p waveform can vanish. We reasoned that such changes of needle position and ECG waveform are anatomically linked. This is because when the zebrafish is positioned with its abdominal side up, the atrium is located slightly toward the right of the center body line^20^. Therefore, extra attention should be paid to properly positioning the needle electrodes at the appropriate sites, which might require some practice when performing zebrafish ECG recording for the first time. We believe that by following the protocol provided in this study, any researcher can carry out successful ECG recording on adult zebrafish.

As for sedating zebrafish during ECG recording, anesthetic agents are recommend to immobilize the fish and relieve its discomfort^21^. To date, MS-222 is the only FDA-approved anesthetic for fishes, which is a sodium channel blocker and has been reported to affect zebrafish heart rate in numerous studies^6,22^. However, we demonstrated that MS-222 not only significantly decreased the zebrafish heart rate but could also result in zebrafish mortality after 10-minute sedation. To support ‘3R’ efforts, i.e., the refinement, reduction and replacement of animal studies, we suggest the combined formula of MS-222 and isoflurane to provide safer sedation for adult zebrafish. While the heart rate could generally decrease after prolonged sedation under the combined MS-222 and isoflurane formula, we found that all zebrafish could be revived even after 30 minutes of prolonged sedation. Most importantly, we demonstrated that the heart rate of adult zebrafish could remain in the normal range within the first five minutes of sedation. We also found that during testing the effect of veratridine, in comparison to MS-222 only, the combined formula of MS-222 and isoflurane could effectively prevent zebrafish death (data not shown). Together, these findings indicated the advantage of the combination formula of MS-222 and isoflurane, which should be especially useful when applying ECG methods for cardiovascular drug screening^3,5,23^.

Regarding other zebrafish ECG methods, *in vitro* recording of adult zebrafish heart ECG has been reported, but such *in vitro* ECG may not capture the integrated responses in the whole living organism in real time^23^. In a preliminary experiment, we have tested our *in vivo* system for recording mouse ECG^24^ (data not shown). We found the system could indeed be employed to monitor heart rate variability in mouse. However, the system may not yet be suited for continuous long-term monitoring of mouse ECG with the current configuration; therefore, we plan to conduct further study to improve the system for mouse ECG monitoring and drug screening.

We tested the zebrafish ECG system in regard to recording the QT interval in adult zebrafish under the influence of selected cardiovascular medications. The QT interval is measured between the onset of ventricular depolarization and the end of the repolarization. Due to its heart-rate dependence, the QT interval may be altered by various pathophysiologic and pharmacologic influences. Thus, the QT interval is often corrected for heart rate, which is annotated as QTc interval. QTc prolongation has been shown to be associated with various forms of tachycardia, and it may also arise from drugs that delay cardiac repolarization. It shall be noted that the Fridericia formula was used in this study to calculate QTc. Although the Bazett formula is the most widely used correction method in clinical practice, the Fridericia formula is recommended by the U.S. Food and Drug Administration (FDA) for clinical trials on drug safety^25^.

QT prolongation can promote lethal arrhythmias such as torsade de pointes (TdP) and have severe adverse effects on patients at risk. We tested two drugs with different mechanism of action that commonly cause prolonged QT in human, and both drugs could induce QTc prolongation in adult zebrafish. We also tested the class I antiarrhythmic agent quinidine, which induces TdP in only 1–3% of quinidine treated human patients. While classical TdP was not observed in quinidine-treated zebrafish, we did observe significant drug-induced high-degree AV blocks at 500 uM quinidine (Supplemental Fig. 3). Thus, the ECG system presented in this study has the potential to expedite the use of adult zebrafish for cardiac toxicity screening.

Although the combination of MS-222 and isoflurane had a weaker proarrhythmic effect than that of MS-222 alone and prolonged the survival time of adult zebrafish over that with MS-222 alone, we noted that this combination did not eliminate gill movement completely during the experiment, which might have interfered with the ECG recording. Other anesthetic agents might stop the gill movement during ECG recording, but they can also induce hypoxia and bradycardia^3,4^. To mitigate such detrimental effects, a perfusion system has been proposed to maintain the zebrafish viable during ECG recording. Moreover, it is possible to apply a low-pass filter in data processing to reduce the signal noise from gill movement and perfusion pulse, which are the most significant sources of noise of zebrafish ECG^3,7,26^. In this study, we adapted the Biosppy toolbox in Python which had been modified to process adult zebrafish ECG signals and to filter the noise form gill movement. However, we plan to continue to modify the system and data processing technique to improve the ECG signal quality. Until such improvements are made, the current ECG system might serve as a convenient and the most economical zebrafish ECG system for the research community.

In summary, the three-needle electrode probe and *in vivo* ECG recording system described in this study could be an entry-level ECG assay platform for teaching and research in zebrafish laboratories. The system could also be used for small-scale cardiovascular drug research or forward genetic screening. We consider this ECG assay system to have promising potential to promote educational and translational applications of zebrafish model systems in future heart research.

## Materials and Methods

### Zebrafish

Adult zebrafish (wild type AB strain) aged from 10 to 12 months (body lengths approximately 3–3.5 cm) were used in this study. All zebrafish were reared in specialized tanks (AZOO, Taiwan) with a circulating water system at a density of approximately 30–50 fish per 10 liters of water under standardized conditions as reported previously^27^. All animal protocols in this study were reviewed and approved by the Experimental Animal Care and Use Committee of National Tsing Hua University, Hsinchu, Taiwan (approval number: 10048). All experiments were performed in accordance with relevant guidelines and regulations.

### Anesthetics preparation

We followed a previously described protocol to sedate adult zebrafish for ECG recording^3^. Tricaine (Sigma, USA) was dissolved in distilled water to a final concentration of 2,000 parts-per-million (ppm) as a stock, and the pH value was adjusted to 7.2 with sodium hydroxide (Sigma-Aldrich). Isoflurane (Baxter, USA) was dissolved in absolute ethanol to create a stock solution of 100,000 ppm (isoflurane: ethanol = 1:9) and maintained in a brown glass bottle. Stocks were all stored at 4 °C. For combined use of tricaine and isoflurane, each stock solution was added to the fish tank to the prescribed final concentration immediately before use.

### Electrode probes

In preparing the electrode probes, we tested three types of electrode materials: tungsten filament, stainless steel and silver wire. During testing, we found that the tungsten filament could be made very thin (25 μm) and that it inflicted minimal injury to the zebrafish when inserted through the dermis. However, the tungsten filament was overly soft and thus easily deformed. The 100% silver needle probes were also easily deformed when inserted directly into the fish dermis. We also tested a silver needle probe composited with 70% silver (350 μm) and obtained results similar to those obtained with the stainless steel probes. Last, we tested stainless steel probes (330 μm) and found they were the most suitable type of electrodes for our ECG system; these probes are also widely used in electrophysiology research^3,28^. The general characteristics of the stainless steel were its relatively high conductivity and high tensile strength, enabling the needle to penetrate the fish dermis easily and provide strong electrical signals. The design of the needle electrode set is illustrated in Supplemental Fig. 1A, and its construction is described in results.

### ECG kit

The ECG signals were recorded using a commercial ECG kit, which uses an USB cable to connect the instrument to a computer that runs the packaged software “ECG Recording System” provided by the manufacturer (model No. EZ-BIO-01-S1-E, Ez-Instrument Technology Co., Taiwan; www.ezinstrument.com/en). The ECG kit records at a data rate of 600 SPS with a digital low pass filter at 80 Hz. The kit also contains an AC-line filter at 60 Hz to eliminate line noise. The kit’s signal-to-noise ratio evaluation was shown in Supplemental Fig. 2A.

### ECG signal processing

The ECG signal of adult zebrafish was processed using Biosppy toolbox^29^. The ECG R-peak segmentation algorithm implemented was based on the literature^30,31^. Specifically, a finite impulse response (FIR) band-pass filter, cut off frequency set between 3 and 45 Hz, was used to filter raw ECG signals. The filtered signals were then passed through additional low-pass filter and high-pass filter to remove low and high frequency noise, and then we calculated its first derivatives^32^. We used window of moving average for smoothing and removing 50 Hz power and muscle activity noise (Supplemental Fig. 2B). The QRS detection algorithm was based on the literatures^33^, and the thresholds were set for detecting and segment every P waves, QRS complexes and T waves as templates. After that, the templates were filtered using moving average window (N is around 10). We averaged all templates of each ECG recording as the final ECG template (Supplemental Fig. 2C). This template was then used as the reference wave.

### Drug treatment

The retro-orbital injection method was used to administrate isoproterenol and verapamil into the zebrafish during ECG recording. The procedure followed a previously described protocol with some modifications^34^. Briefly, the injection site was the retro-orbital venous sinus, which is located beneath the zebrafish eyeball. A 26S-gauge Hamilton syringe filled with the prescribed drug was positioned above the anesthetized fish’s eye at the 7 o’clock position at a 45-degree angle to the fish body. After insertion of the needle into the eye socket, the drug was gently injected without moving the fish during continuous ECG recoding. For amiodarone drug testing, adult zebrafish were pre-exposed to the chemical by immersion in a water bath containing 100 μM amiodarone for one hour.

### Statistical analysis

Data were processed by SigmaPlot v.10 and expressed as the means ± SEM (standard error of the mean). Statistical significance was determined by the Student *t*-test. Significant differences were assessed at *p* values of <0.05 (*) and <0.001 (**). QTc intervals were normalized to heart rate using the standard Fridericia’s formula:

$${\rm{QTc}}=\mathrm{QT}/\sqrt[3]{{\rm{RR}}}.$$

## Electronic supplementary material

Supplementary Information

## Data Availability

The datasets generated and analyzed during the current study are available from the corresponding author on reasonable request.
