# Supplementary material for: Development of a rapid and economic in vivoelectrocardiogram platform for cardiovascular drug assay and electrophysiology research in adult zebrafish
Source: Sci Rep. 2018 Oct 30;8:15986. doi: 10.1038/s41598-018-33577-7 (PMC6207748; doi:10.1038/s41598-018-33577-7)

Development of a rapid and economic *in vivo* electrocardiogram platform for cardiovascular drug assay and electrophysiology research in adult zebrafish

Min-Hsuan Lin[1], Huang-Cheng Chou[2], Yu-Fu Chen[3], Wangta Liu[4], Chi-Chun Lee[2], Lawrence Yu-Min Liu[1,5,*] and Yung-Jen Chuang[1,*]

[1]Department of Medical Science & Institute of Bioinformatics and Structural Biology, National Tsing Hua University, Hsinchu, 30013, Taiwan

[2]Department of Electrical Engineering, National Tsing Hua University, Hsinchu, 30013, Taiwan

[3]Department of Medical Science, National Tsing Hua University, Hsinchu, 30013, Taiwan

[4]Department of Biotechnology, College of Life Science, Kaohsiung Medical University, Kaohsiung, 80708, Taiwan

[5]Division of Cardiology, Department of Internal Medicine, Hsinchu Mackay Memorial Hospital, Hsinchu, 30071, Taiwan

* Corresponding author: yjchuang@life.nthu.edu.tw and drlawrenceliu@gmail.com

Supplementary Information

Supplemental Figure 1. The design of the three-point needle electrode probe

(A) Scheme of the ECG recording platform, with the three-needle electrode probe connected to the ECG kit and a computer pre-loaded with software.

(B) The electrode probe set consists of two detecting electrode probes with a syringe holder and one grounding electrode.

(C) Close-up view of the detecting electrode.

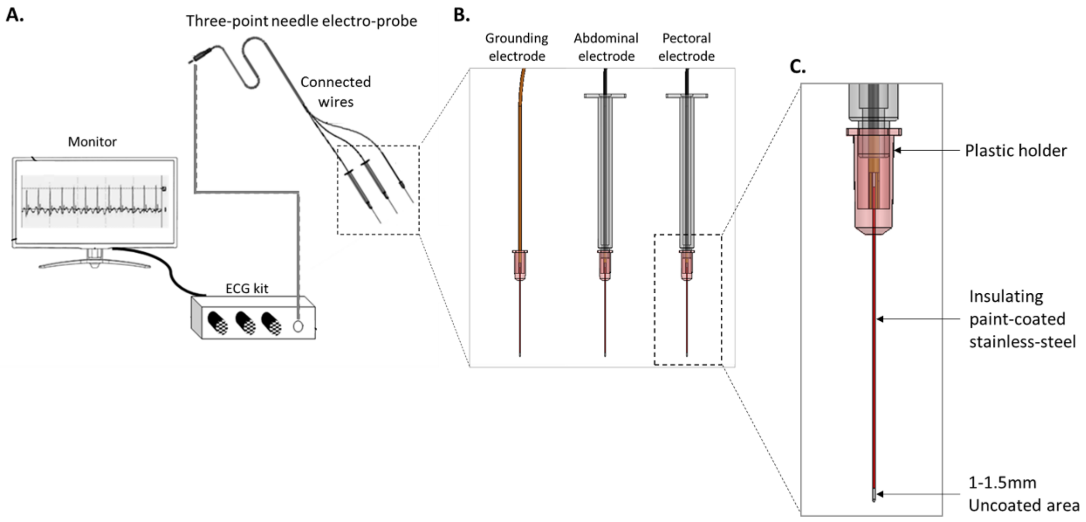

Supplemental Figure 2. The results of Signal-to-noise ratio evaluation for EzInstrument's ECG Kit and data analysis method.

(A) The instrument's SNR evaluation was completed with grounding the reference probe to the ground and inputting with a sine wave from a functional generator to another probe. And then, the output voltage was measured for comparison with the noise level (the maximum deviation), also measured from the oscilloscope at the same time. The range of adult zebrafish heart rates (50-200 bpm) analyzed in this study represents the frequency ≤ 3 Hz.

(B) The raw signal (black line) and de-noise signal (blue line).

(C) Overlay of extracted traces were plotted as the gray color. The black line was the average of ECG.

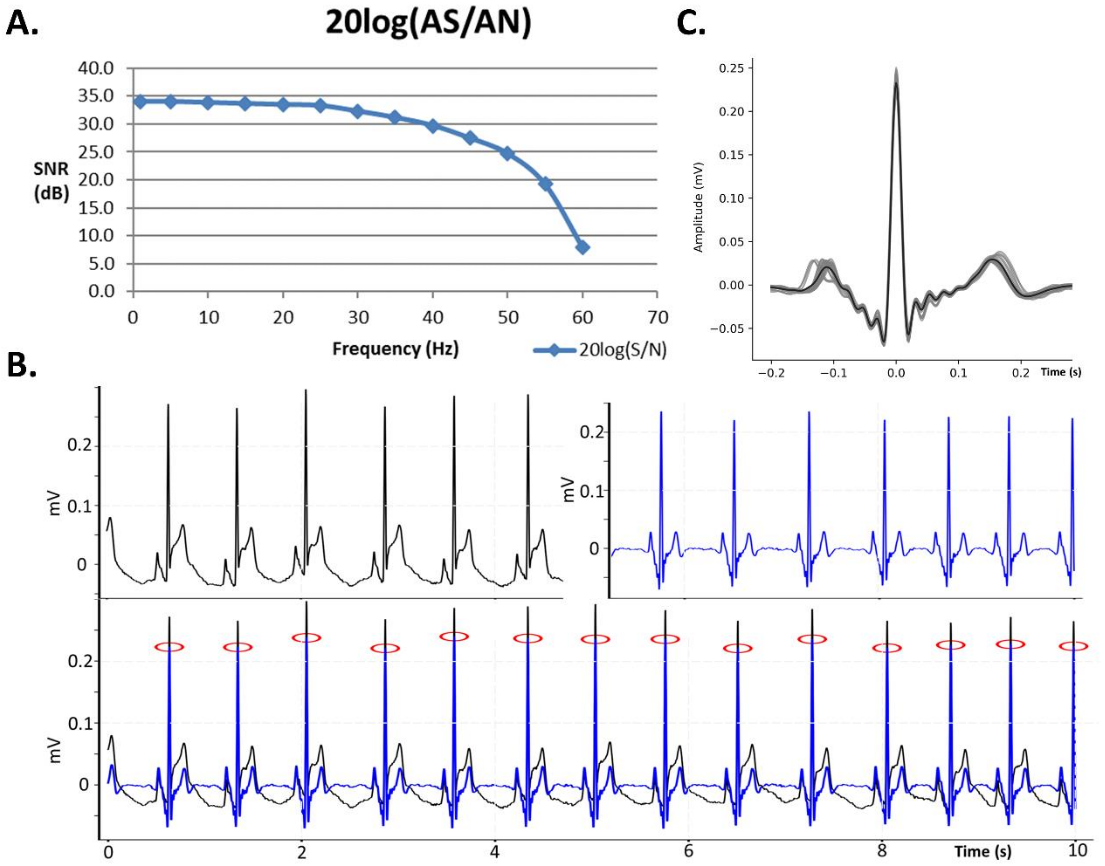

Supplemental Figure 3. Quinidine induced high-degree AV blocks

(A) Second degree AV block (2:1 block) was observed after quinidine (500 µM) was injected.

(B) High-degree AV block was also observed. More than two consecutive P waves were blocked and there was significant AV dissociation.

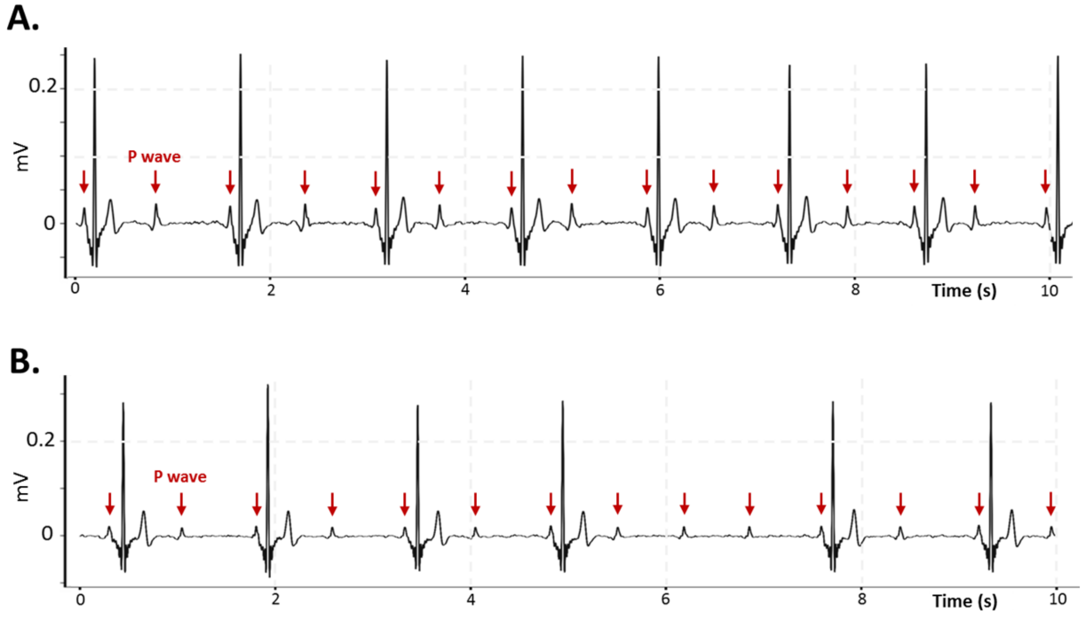

Supplement: Supplementary file 1 — Supplementary Information [file 41598_2018_33577_MOESM1_ESM.pdf]
